# Rain drives foraging decisions of an urban exploiter

**DOI:** 10.1371/journal.pone.0194484

**Published:** 2018-04-11

**Authors:** Matthew Chard, Kris French, John Martin, Richard E. Major

**Affiliations:** 1 School of Biological Sciences, University of Wollongong, Wollongong NSW, Australia; 2 Royal Botanic Gardens & Domain Trust, Sydney NSW, Australia; 3 Australian Museum Research Institute, Australian Museum, Sydney NSW, Australia; University of Sydney, AUSTRALIA

## Abstract

Foraging decisions tend to drive individuals toward maximising energetic gains within a patchy environment. This study aims to determine the extent to which rainfall, and associated changes in food availability, can explain foraging decisions within a patchy urbanised landscape, using the Australian white ibis as a model species. Ibis density, food consumption rates and food abundance (both natural and anthropogenic) were recorded during dry and wet weather within urban parks in Sydney, Australia. Rainfall influenced ibis density in these urban parks. Of the four parks assessed, the site with the highest level of anthropogenic food and the lowest abundance of natural food (earthworms), irrespective of weather, was observed to have three times the density of ibis. Rainfall significantly increased the rate of earthworm consumption as well as their relative availability in all sites. Overall, these density and consumption measures indicate that anthropogenic derived foods, mainly from direct feeding by people, explain the apparent distribution of ibis across urban parks. However, there was evidence of prey-switching when the availability of natural foods increased following rainfall, perhaps reflecting selection of particular nutrients.

## Introduction

Foraging theory suggests that foragers maximise energetic gains by selectively exploiting patches rich in resources and by minimising foraging time in poor patches [1]. To achieve this, foraging individuals must be able to (1) recognize patch boundaries, (2) estimate patch rewards, and (3) decide when to leave a patch [2]. Within a particular environment, an individual can modify their behaviour after assessing available resources [3], risks of predation [4], energetic costs associated with foraging [1,5] or by associations with past experience (foraging memory) [6] within the limitations imposed by their capacity to search [7,8]. Interestingly, few studies have explored how climatic processes may alter the foraging envelope.

Urbanisation has provided a range of adaptable species with the capacity to exploit novel food resources [9–14]. Urban environments are characterised by broad areas of built structures, impervious surfaces, and fragmented areas of greenspace [15], which are characterised by consistency, with well-watered parks and gardens supporting plant growth and foraging opportunities [16]. Foraging by urban birds is influenced by the presence and size of remnant greenspace [17,18], intraspecific competition [19,20]; predation [21,22], the structure and floristic attributes of planted vegetation [23–26], and supplementary feeding by humans [20,27,28]. Urban greenspaces often have high predictability of food and water [9,29,30]. Therefore, higher densities of foragers, such as birds, are predicted to be supported within food-enriched patches [16,30,31].

While not previously investigated in the urban context, changing levels of rainfall at sites also affects the foraging behaviour of bird species [32–34]. Studies investigating animal responses to climatic variation often focus on wider ecological responses such as variation in population size [35–37], community composition [38–40], individual movements [41,42], reproductive output [43] and habitat selection [42,44] through measuring climatic variation at national and regional scales [45,46]. Rainfall significantly influences reproduction [36,47–49], population distribution and movement [50–52] as well as energy allocation [32]. However, there has been a small body of work investigating the influence of local rainfall events on behavioural decisions [33,53,54] and none in urban habitats.

Rainfall can be an important driver of food availability for waterbird species [48,55] stimulating breeding events [56,57]. While most literature focuses on food availability in natural habitats of waterbirds, it is predicted that increases in natural food availability, due to rainfall, will also occur in areas of greenspace distributed within an urban setting [58]. Urban-adapted species may recognize the physical cue of rain to indicate ameliorated and increased natural food acquisition within certain patches scattered throughout the urban matrix in the same way that they respond in natural environments. Alternatively, rainfall may reduce some food items, such as a reduction in human-based foods as people move indoors during rain [58].

The Australian white ibis (*Threskiornis molucca*, hereafter referred to as ibis), has recently shifted its distribution toward the east coast of Australia in association with a decline in environmental water availability [55,59]. The population of ibis within urban landscapes has increased dramatically over the last 40 years [60–62]. The high abundance of ibis in urban areas has led to them being considered as pests due to social, economic and environmental problems [63], including the possible spread of pathogens [64] and threats to aircraft safety [65]. While some studies have investigated movements in cities [66,67], there remains a need for studies investigating the foraging behaviours and habitat choices of birds within the urban setting. The choice of natural versus anthropogenic food can have serious implications for avian health, as waste-supplemented diets have been shown to be nutritionally inadequate for normal chick development [68–70]. Ibis are known to forage on both anthropogenic and natural foods in urban landscapes, but the mechanism which drive these foraging decisions is currently unknown.

The aim of this study is to understand foraging decisions of an urban bird which might explain movements between patches, particularly in association with rainfall. Firstly, we assessed how abundance, and consequently the density, of ibis changes between sites within a patchy environment before and after rainfall events. Secondly, we measured food consumption rates of ibis within each site before and after rainfall events, to identify changes in ‘profitability’ of each site in terms of prey capture rates. Linking these two findings we determined whether spatial and weather-driven variation in specific prey items could explain the observed foraging behaviour. Lastly, the influence of anthropogenic food items was investigated to further understand the underlying mechanisms behind ibis foraging choices.

## Methods

The study was conducted in four inner city parks within a 1.5 km^2^ area in the Sydney central business district (CBD), Australia. Each park was chosen on the basis that it regularly supported foraging ibis [61]. The anthropogenic exposure is dynamic, changing in intensity with events, seasons and weather.

Belmore Park (33°52′53″S 151°12′28″E) is a small (2.5 ha) park that experiences a high daily flow of human traffic, presenting opportunities for birds to consume anthropogenic food via direct feeding or scavenging. There are also natural foraging opportunities in garden beds and within grassed areas but ibis have not been observed breeding or roosting in Belmore Park.

Hyde Park (33°52′24″S 151°12′41″E, 16.2 ha) is divided in two by a road; the south side contains a pool where ibis can drink and bathe, the north side contains a café and three water fountains. Ibis forage within the garden beds and grassed areas, as well as scavenge from people consuming food, mostly during the lunch hours. Ibis have been observed to nest in the palm trees in the northernmost part of the park.

The Domain (33°52′6″S 151°12′53″E, 34 ha), is separated by a minor road into two sections (between which ibis frequently walk), and experiences high human traffic daily. The Domain is used daily by people for exercise and relaxation. Ibis are often observed foraging naturally, but scavenging does occur around a café and from small numbers of people having lunch. Ibis breed in a discrete palm grove.

The Royal Botanic Garden (33°51′50″S 151°13′1″E, hereafter referred to as Garden; 30 ha), contains an assortment of garden beds and lawns and contains water features, buildings and pathways. Ibis are actively discouraged (by nest removal) from nesting, yet nesting occurs within palms on the periphery of the Garden. Ibis forage naturally within the extensive garden beds and grassed areas, and they also scavenge anthropogenic food from café diners and picnickers.

Permission to conduct the observational study was granted by the City of Sydney and the Royal Botanic Garden and Domain Trust. Animal ethics was approved by the NSW Office of Environment and Heritage (100913/04). All sampling procedures were specifically approved as part of obtaining the field permit.

### Ibis abundance and forging

Data on ibis numbers and foraging rates were collected on 20 days between 5/05/2015 and 2/09/2015. As the study aimed to determine how rainfall influences ibis foraging decisions, weather forecasts from the Bureau of Meteorology (www.bom.com.au, S1 File) were used to identify rainfall events. Daily rainfall data from three nearby weather stations (Observatory Hill, Centennial Park and Royal Botanic Garden—all within 4 km) were averaged to calculate an approximation of the daily rainfall experienced across the Sydney CBD. A rainfall event was defined as any period for which the mean daily rainfall exceeded 2 mm, and surveys occurring within two days subsequent to a rainfall event were defined as ‘wet’. Surveys were defined as ‘dry’ if less than 2 mm of rainfall was received for at least two consecutive days before the survey. All four parks were assessed on each survey day, and the order in which the parks were visited was randomised.

In order to determine the total abundance of ibis, each park was surveyed in the morning between 7am and 10am by traversing the full extent of each park. During each survey the number of ibis located within the confines of each site was recorded and the entire area could easily be searched with little chance of missing birds. Each site was visited a total of twenty times, with ten ‘wet’ and ten ‘dry’ surveys. Due to the differing size of each site, abundance was divided by the area of the greenspace to provide the density, expressed as ibis per hectare. Areas of greenspace were determined using a satellite mapping tool (SIXmaps: www.maps.six.nsw.gov.au). A two-way analysis of variance (ANOVA), followed by Tukey’s HSD post-hoc tests, was used to analyse the difference in ibis density between sites during ‘wet’ and ‘dry’ days.

### Earthworm consumption rate and abundance

In order to determine the prey consumption rate of “naturally” foraging ibis, each park was surveyed in the morning between 7 am and 10 am. An ibis was considered to be naturally foraging if it was fossicking, jabbing, probing or pecking at substrate or handling prey items [71,72]. All other behaviour was considered non-foraging and was not scored.

The foraging behaviour of individual ibis was observed at a distance of at least 10 m. A total of 30 min of continuous foraging behaviour from a number of ibis was observed during ten wet and ten dry periods. Individual ibis were observed for at least 5 min and no more than 10 min. This resulted in 10 hours of observational data at each site.

The number of successful consumptions of natural prey items was recorded, indicated by a backward jerking motion of the head which propelled the food item from the tip of the beak to the gullet. This was then divided by 30 min to provide a consumption rate per minute. A two-way ANOVA was used to analyse the total number of successful consumptions of prey items per minute during ‘wet’ and ‘dry’ days.

Further, if the prey item consumed by the focal ibis was able to be identified as an earthworm, then this was also recorded. These data were used to infer what proportion of the ibis natural prey consisted of earthworms. Again, the data were separated into ‘wet’ and ‘dry’ categories and analysed with two-way ANOVA.

The relative abundance of earthworms at each park was measured between 7–10 am. A patch of 0.5 ha was selected in each site where ibis had been observed foraging naturally in both ‘wet’ and ‘dry’ conditions (see foraging data, above). An aqueous solution (4 g/L^-1^) of powdered chlorinated trisodium phosphate (TSP), a skin irritant, was applied to the soil to drive worms from the sub-surface layer [73]. A pilot study identified this irritant as very effective while not damaging the worms or the grass. Within the selected patch, 4 litres of this solution was poured into a randomly placed 1 m^2^ quadrat. Three quadrats were measured at each site during three separate ‘dry’ and ‘wet’ weather conditions. Earthworms which evacuated the substrate were collected, counted, washed with water and replaced on the substrate close to, but not within, the quadrat.

### Anthropogenic food assessment

The amount of anthropogenic food accessible to ibis within urban parks was estimated using a combination of quantitative measures, assessed by three variables. Firstly, the number of people who brought food items into each site was counted on four days during the lunch period (12pm– 2pm). This was done by a quick walk through each park counting any person that had some form of consumable item. This count was categorised as ‘lunchers’ and expressed as the density of lunchers per hectare within each urban park. Secondly, throughout the study we noted any occasion an individual person was specifically observed feeding large quantities of food to ibis, as opposed to feeding scraps of food from their own lunch. This count was categorised as ‘dumpers’ and used to assess how much targeted feeding was taking place within each urban park. We spent approximately equal time in each park, so survey effort was comparable among parks. Thirdly, as ibis have been observed removing waste directly from bins the number of bins within each park was counted and expressed as a density of bins per hectare within each site.

## Results

### Ibis abundance and foraging

**Fig 1:**
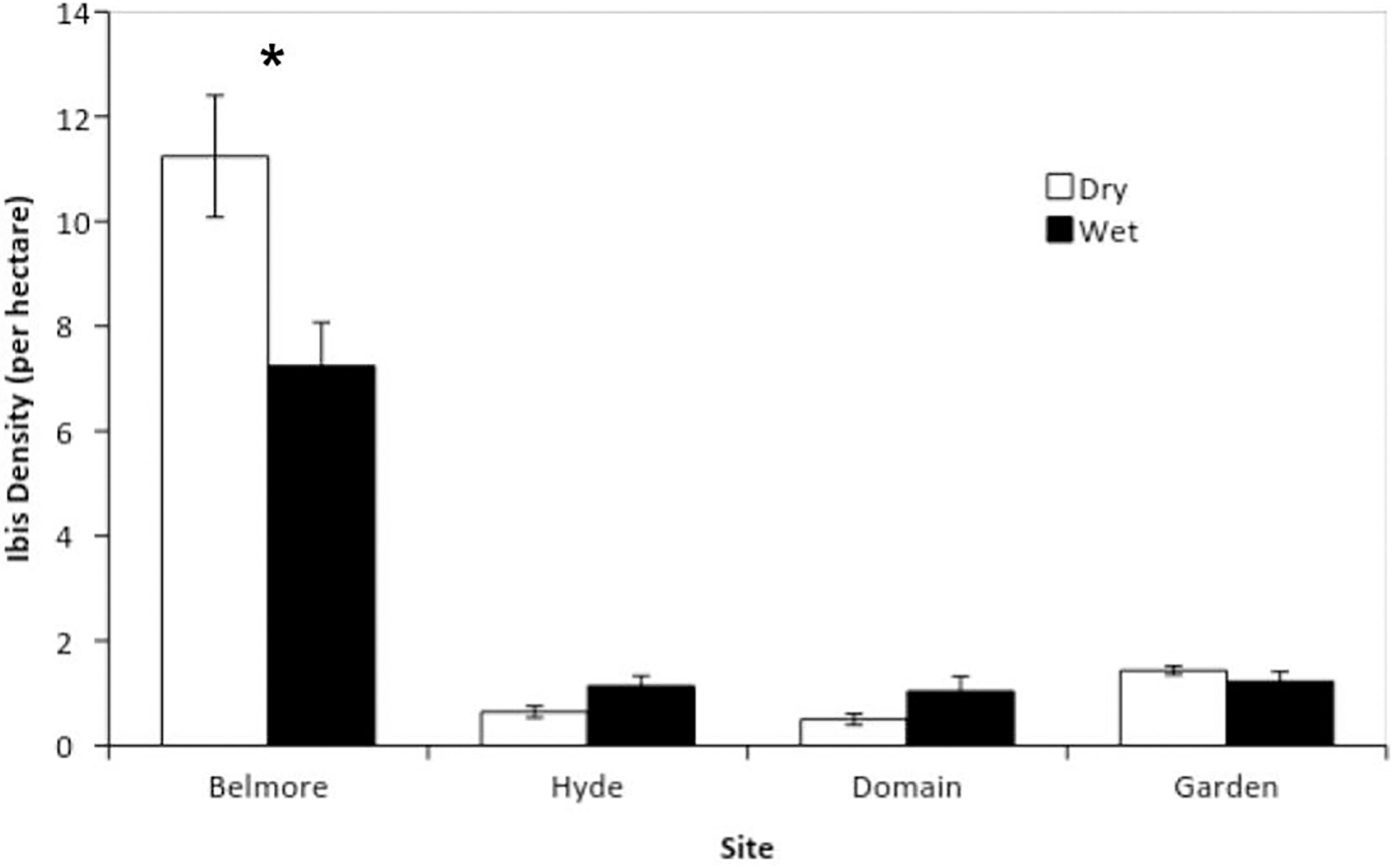
Mean (±SE) ibis density in four parks in the Sydney central business district. Each park was surveyed on 10 ‘dry’ (no rain) and 10 ‘wet’ (during rainfall) days. Density measures were calculated using the maximum observable area of each site and expressed as the number of birds per hectare. *denotes significance at p < 0.05.

The effect of rainfall on the density of ibis varied significantly between sites (interaction; F_1,3_ = 8.28, p < 0.0001). In Belmore Park, the density of ibis was significantly higher during dry periods than in wet periods (Fig 1). The density of birds did not vary with weather at the other sites although there was a trend for higher densities during wet surveys at both Hyde Park and the Domain (Fig 1). Ibis density was significantly higher in Belmore Park than the other three sites, irrespective of the weather (Fig 1).

**Fig 2:**
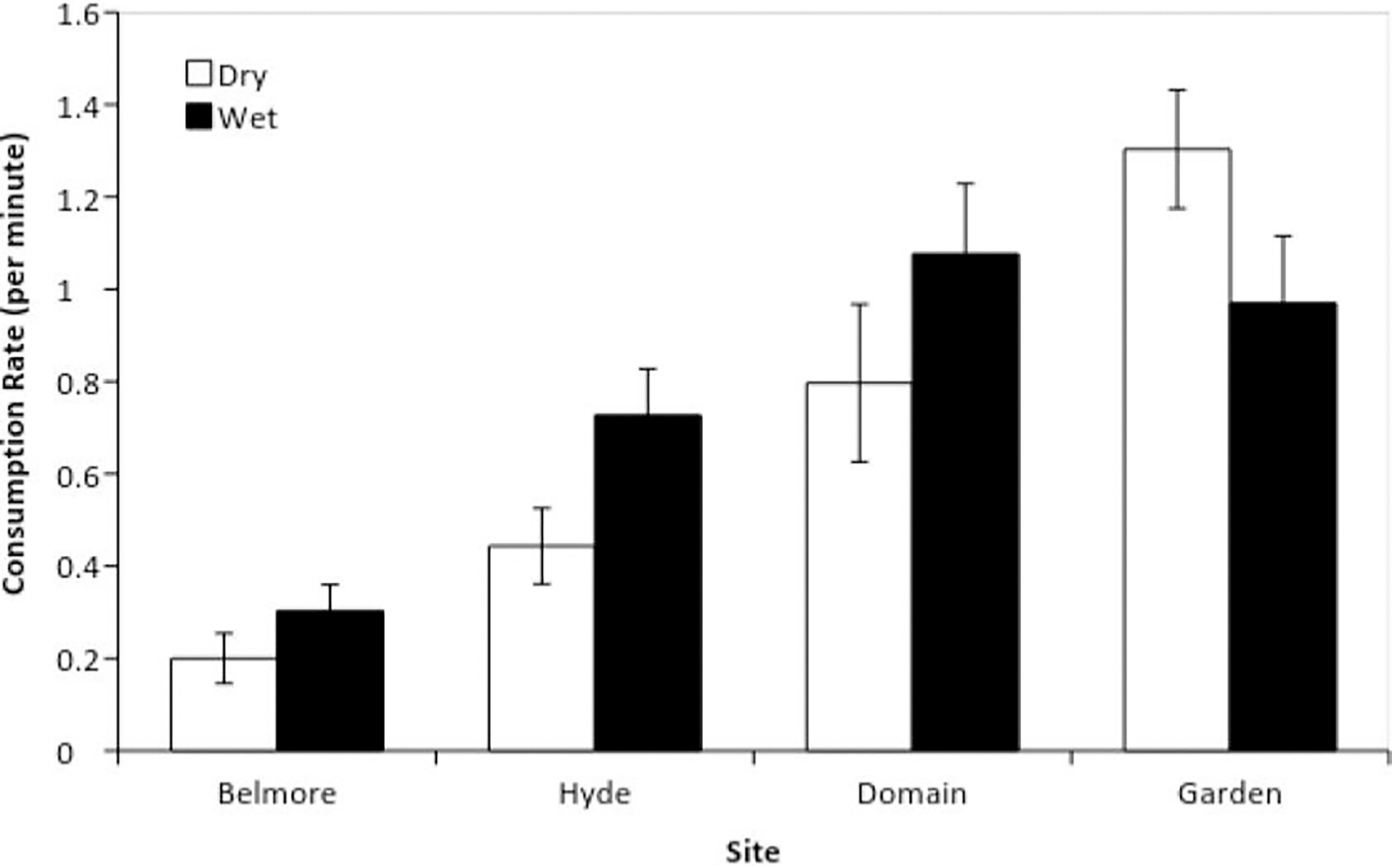
Mean (±SE) consumption rates of natural food items by ibis in four parks in the Sydney central business district. Each park was surveyed on 10 ‘dry’ (no rain) and 10 ‘wet’ (during rainfall) days.

The effect of rainfall on the consumption of natural prey items differed between sites (interaction; F_1,3_ = 3.03, p = 0.035), however Tukeys tests could not distinguish differences between consumption rates during wet and dry periods for any site. The number of prey items consumed by ibis ranged from 0.2 to 1.3 min^-1^ (Fig 2). The highest consumption rate of natural foods was recorded at the Garden (1.3 min^-1^) during dry periods. Ibis tended to consume more prey items on ‘wet’ days at the Domain and Hyde Park. The lowest proportion of natural foraging was recorded in Belmore Park. The effect of rainfall across each site was not found to significantly influence consumption rates.

### Earthworm consumption rate and abundance

**Fig 3:**
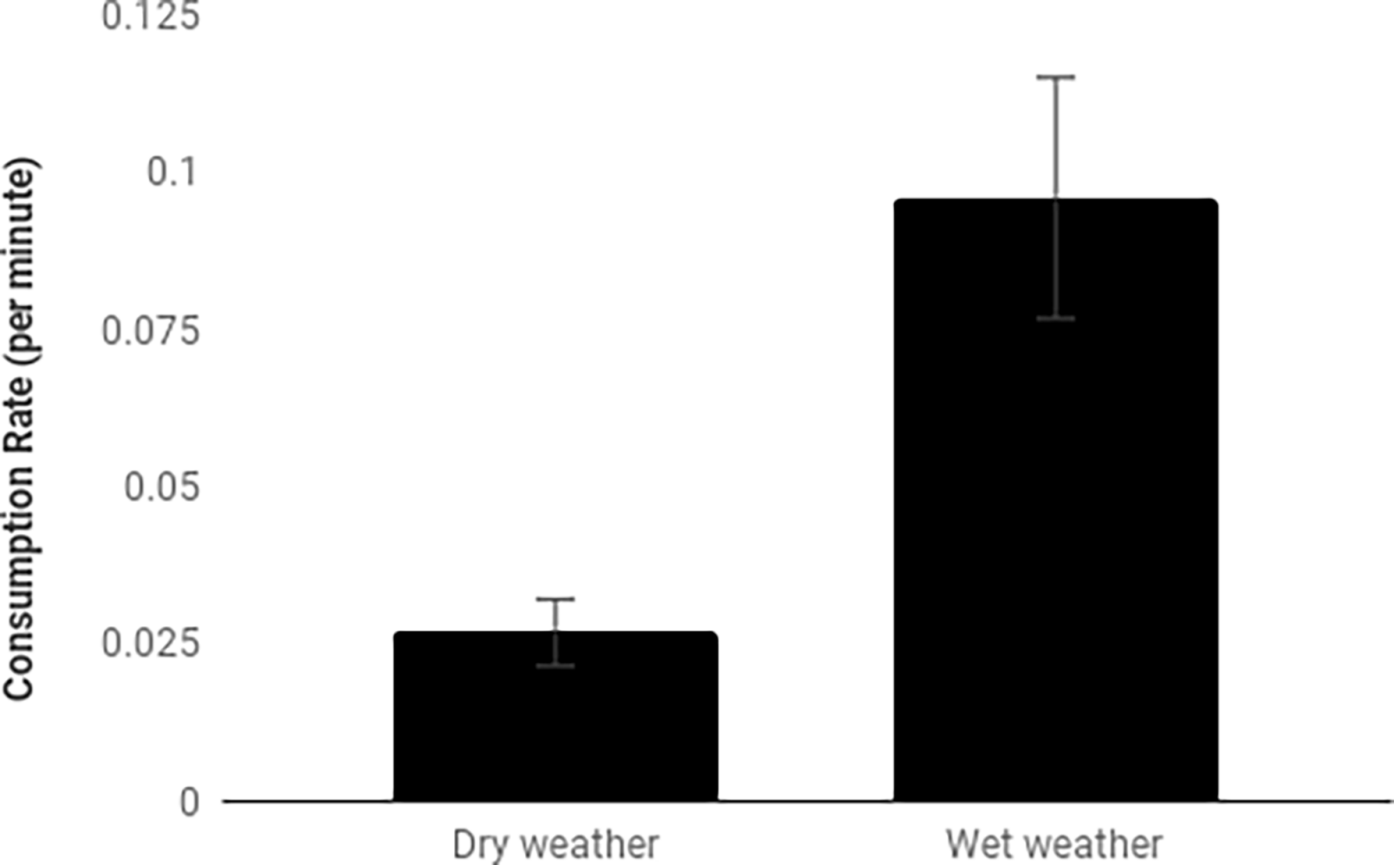
Mean (±SE) rate of earthworms consumed by naturally foraging ibis in four urban parks in the Sydney central business district. Sites were surveyed on 10 ‘dry’ (no rain) and 10 ‘wet’ (during rainfall) days for 30 min each day and pooled across sites as there was no significant interaction term.

Ibis consumed significantly more earthworms after rainfall than in dry weather (F_1,3_ = 14.46, p = 0.003). The rate at which ibis consumed earthworms was three times higher during wet weather (Fig 3). There was some indication that the quantity of earthworms consumed differed between sites, but this was not significant (F_1,3_ = 2.67, p = 0.054). There was no evidence of a significant interaction between sites and rainfall (F_1,3_ = 14.46, p = 0.710).

**Fig 4:**
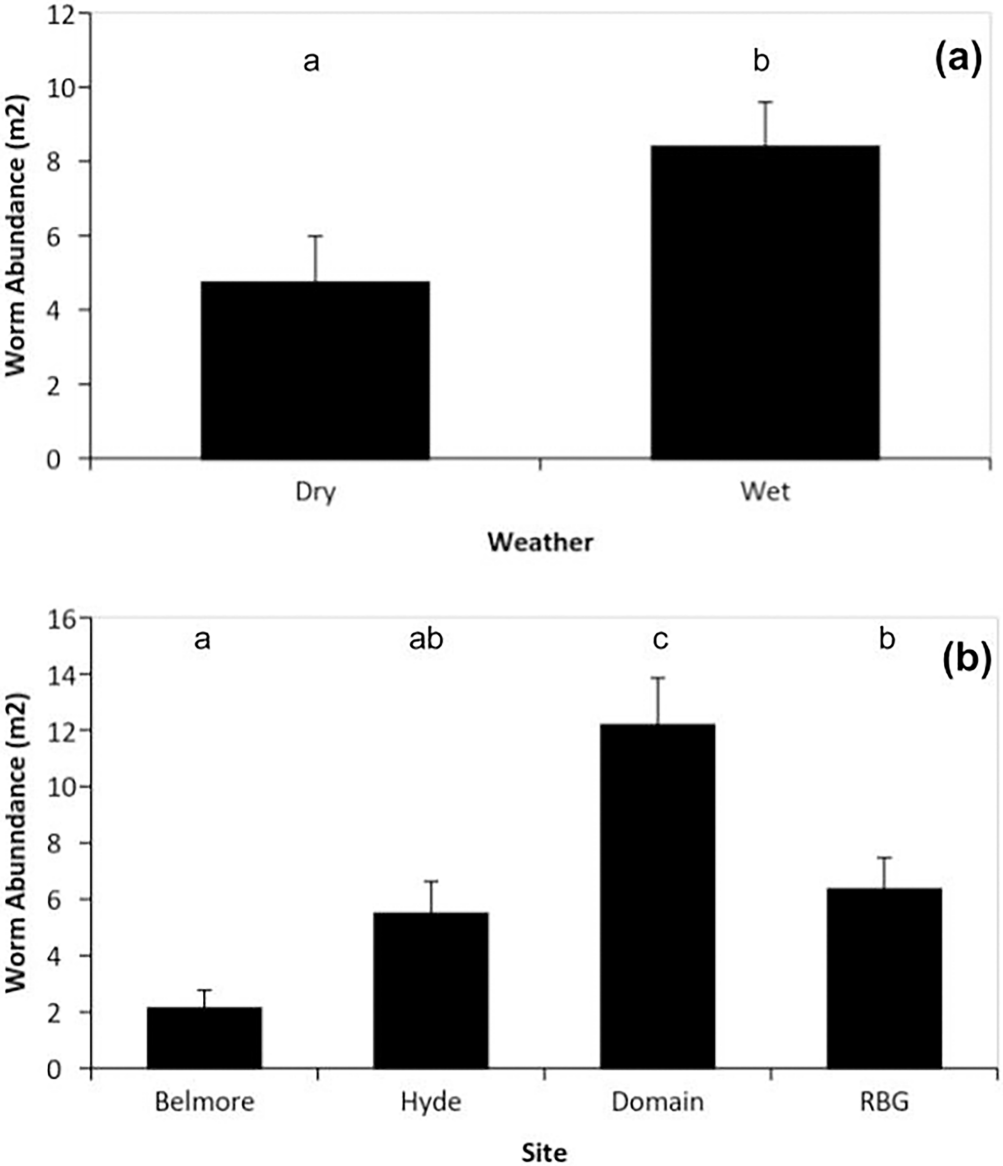
Mean (±SE) abundance of earthworms within a 1-m^2^ quadrat at four Sydney parks measured on three ‘dry’ (no rain) and three ‘wet’ (during rainfall) days at each site: (a) worm abundance in different weather conditions; (b) worm abundance in different sites. Tukey’s HSD significance indicated by letters above each site; sites not sharing the same letter are significantly different.

After rainfall events the abundance of worms significantly increased across all sites from 4.7 worms/m^2^, during dry conditions, to 8.4 worms/m^2^ after rainfall events (F_1,3_ = 14.68, p < 0.0015; Fig 4A). The abundance of worms in the soil varied significantly between the four urban parks (F_1,3_ = 19.23, p < 0.0001), with the Domain having a significantly higher density than the other sites and Belmore Park having the lowest density (Fig 4B). There was no significant interaction between earthworm abundance and site (F_1,3_ = 0.25, p = 0.859).

### Anthropogenic food availability

Indices of anthropogenic food provisioning were not consistent amongst sites but overall, Belmore Park and Hyde Park appeared to have the highest anthropogenic food availability. The level of food consumption by people during the lunch period was highest at Hyde Park while the density of bins was highest at Belmore Park (Table 1). Deliberate bird feeding by people (dumpers) was only observed at two of the parks; at Belmore Park, where food dumping occurred on 15 occasions (60% of surveys) and at Hyde Park on two occasions.

**Table 1:** Relative availability of anthropogenic food within four parks in Sydney. Three variables were measured: the density of bins; the number of occasions in which people were observed to deliberately feed ibis (‘dumpers’); and the number of people consuming lunch within the park (‘lunchers’, n = 4).

Site	Bins (per ha)	Dumpers	Lunchers (per ha)
**Belmore Park**	6.4	15	2.2 (± 0.7)
**Hyde Park**	3.8	2	6.5 (± 1.1)
**Domain**	1.0	0	3.0 (± 0.7)
**Garden**	2.3	0	1.3 (± 0.4)

## Discussion

The abundance of ibis differed in response to rainfall across the four sites surveyed. Ibis density decreased after rainfall events at Belmore Park, confirming results from 7 years of records [58]. Changes in abundance during, and immediately after, rainfall indicates that rainfall may trigger behavioural responses of ibis within the urban environment. Ibis may perceive that there is decreased food availability in certain patches, while increased food availability in other patches because direct feeding from humans probably decreases during rainfall events, due to people being less likely to spend time outside. Ibis appear to adjust accordingly, presumably due to learned foraging experience and memory [6,74]. While ibis density decreased in Belmore Park in association with rain, it increased in two of the larger sites, Hyde Park and the Domain, though this was not significant. Furthermore, consumption rates of natural prey items and the availability of worms within the substrate increased with rainfall at both Hyde Park and the Domain. Thus, ibis may recognise the external cue of rainfall and link it to increased food abundance in other patches and decide to visit sites rich in natural, rain associated, prey. Interestingly, even during dry periods, these two sites had high densities of worms and high consumption rates by birds visiting these sites, relative to Belmore Park. Thus, in terms of natural foods, these sites were resource rich; although not the preferred sites for ibis.

The consumption of natural foods increased after rainfall events within Belmore Park, Hyde Park and The Domain. The Garden was the exception, with little change in consumption rate with rainfall. In general, in association with rainfall, natural prey became more readily available, presumably because invertebrate species such as earthworms, migrate toward the soil surface and emerge from the substrate perhaps as a result of oxygen depletion [75]. This trend was apparent in both the proportion of worms consumed by ibis, as well as measures of worm abundance. Therefore it may be the case that ibis have a nutritional preference for earthworms, compared to artificial foods, but the acquisition of such prey items is only economically viable after periods of rainfall.

Natural prey items, such as earthworms, are considered nutritionally valuable to ground foraging avifauna and an excellent source of protein [76]. Common mynas, *Sturnus tristis*, had a clear preference for high-protein foods over high-lipid or high-carbohydrate foods within an urban setting [13]. Further, Florida scrub-jays, *Aphelocoma coerulescens*, in suburban environments were observed to discriminate between natural and human foods by showing a preference for natural foods [11]. However, there is an energetic cost associated with the acquisition of natural prey items [77,78] and when handling time of natural foods increases, urban birds have been observed to switch to human-provided foods[11]. A preference for high carbohydrate foods, consistent with human-provided foods, was recently reported for urban ibis [14]. Ibis forage for prey via probing substrate with their long bills [79], and it is speculated that increased soil moisture will also increase the permeability of the substrate in which they feed, allowing a higher rate of prey capture. This phenomenon has been recorded for snipe, lapwings and shanks with localised flooding of grasslands [80]. This study clearly demonstrates that prey switching is occurring within the urban landscape by ibis as capture of natural food sources increases with rainfall. Ibis may recognise rainfall as an environmental cue that individuals will be better off foraging within neighbouring, prey-rich patches because of increased intake rates resulting from greater prey availability and/or decreased handling times.

The density of ibis at Belmore Park was three times higher than the other three sites, suggesting that this urban patch is perceived to be resource-rich [77,78]. Few natural food items are consumed in this park. Further, the abundance of worms was lowest in this patch compared to the other sites. Interestingly, while the availability of natural food items is lower, ibis still spent significant time searching for natural foods within Belmore Park. Individuals are expected to weigh travel distance against resource intake, so we might expect ibis to value Belmore Park as a suitable foraging patch because it might be close to either roosting and/or nesting sites [77,81]. However, ibis do not utilise this site for either roosting or breeding, while roosting and breeding occurs within the other three sites surveyed. Thus, non-resource-based scenarios do not explain why ibis are still deciding to visit this foraging site. The simplest explanation of why ibis are foregoing natural food resources by choosing this park is that they are favouring the acquisition and consumption of anthropogenic food.

Urban ibis are able to supplement their diet with anthropogenic food sources, and it appears that there is a consistent abundance of these foods within this patch. Belmore Park received the highest incidence of food dumping and had a high density of bins per hectare. High levels of predictability and continuous input of food in the urban environment may have also resulted in overmatching, leading to the overexploitation of this rich, urban patch [29,30]. This food-enriched patch appears to have resulted in an extremely high density of ibis within Belmore Park as people regularly visit the park to directly feed ibis, or place waste within bins which the ibis are then able to extract [16,72]. As a result, ibis visiting this park may also forage upon and deplete natural prey items.

If ibis are preferentially choosing to forage where anthropogenic food is potentially more available than natural food, then this could have serious repercussions for the health of the population. Overabundance of food and lack of predators or disease has allowed urban bird populations to increase; this has been shown to decrease individual body condition and life span [29]. Further, urban avifauna may have a trade-off between offspring body condition and clutch size [29] as waste-supplemented diets have been found to be nutritionally inadequate for normal chick development [68–70]. Despite the rise in population within urban areas [61], it is unclear whether ibis are suffering from problems associated with urban diets. Additional research into health, reproduction and chick development is needed to properly assess the implications surrounding the apparent reliance on anthropogenic food sources. This information will be useful to guide future population management within the urban context and the long-term conservation of this native species.

Overall, the results of this study suggests that the high density of ibis in Belmore Park associated with low prey consumption rates, low abundance of natural prey and the prevalence of human-derived foods are consistent with the idea that ibis are choosing waste-supplemented diets. These urban ibis prefer to visit a patch which provides easy, carbohydrate-rich foods sourced from direct anthropogenic feeding or scavenging at bins. Rainfall clearly influences the foraging choices of this species. It would be both scientifically interesting and beneficial to this avian population to investigate how the removal of a factor, such as direct feeding of birds or the introduction of bird-proof bins, could impact foraging decisions within the urban environment.

## Supporting information

S1 File Raw datum from observation experiments of ibis abundance, consumption rates and anthropogenic food sources. Rainfall records also included sourced from the Bureau of Meteorology (www.bom.gov.au)
(XLSX)
